# Perinatal Cat and Dog Exposure and the Risk of Asthma and Allergy in the Urban Environment: A Systematic Review of Longitudinal Studies

**DOI:** 10.1155/2012/176484

**Published:** 2011-11-30

**Authors:** Caroline J. Lodge, Katrina J. Allen, Adrian J. Lowe, David J. Hill, Cliff S. Hosking, Michael J. Abramson, Shyamali C. Dharmage

**Affiliations:** ^1^Centre for Molecular, Environmental, Genetic and Analytic Epidemiology, School of Population Health, University of Melbourne, Melbourne, Vic 3010, Australia; ^2^Murdoch Children's Research Institute, Royal Children's Hospital, Melbourne, Vic 3052, Australia; ^3^Department of Allergy, and Department of Paediatrics, University of Melbourne, Royal Children's Hospital, Melbourne, Vic 3052, Australia; ^4^John Hunter Children's Hospital, Newcastle, NSW 2305, Australia; ^5^Department of Epidemiology and Preventive Medicine, Monash University, The Alfred Hospital, Melbourne, Vic 3004, Australia

## Abstract

*Background*. The literature is contradictory concerning pet exposure and the risk of development of asthma and other allergic diseases. Using longitudinal studies, we aimed to systematically review the impact of pet ownership in the critical perinatal period as a risk factor for allergies in childhood. *Methods*. Medline database was searched for urban cohort studies with perinatal exposure to cats and/or dogs and subsequent asthma or allergic disease. *Results*. Nine articles, comprising 6498 participants, met inclusion criteria. Six found a reduction in allergic disease associated with perinatal exposure to dogs or, cats or dogs. One study found no association. Two found increased risk only in high-risk groups. *Conclusion*. Longitudinal studies in urban populations suggest that perinatal pets, especially dogs, may reduce the development of allergic disease in those without a family history of allergy. Other unmeasured factors such as pet-keeping choices in allergic families may be confounding the association seen in these high-risk families, and further study is required.

## 1. Background

Allergic disease appears to be on the rise worldwide, and although an allergic family history is one of the strongest risk factors for childhood allergy [1], large international studies [2–4] which highlight geographical differences in allergy prevalence, strongly suggest that environmental influences also play a causal role. Although pets are known to aggravate asthma, allergic rhinitis, and eczema in sensitized individuals [5] controversy remains about whether early life pet exposure is a risk factor or a protective factor in their development. Current guidelines issued in Australia [6], the United States [7], and the United Kingdom [8], and by the Global Initiative for Asthma [9] all agree there is currently insufficient evidence to provide any recommendations in relation to pet-keeping in early life and the development of asthma and allergic disease because systematic reviews [10–13] and a meta-analysis [14] have reached different conclusions. Early reviews [10] found pet-keeping increased the risk of sensitization [10] and allergic disease [10, 12] with later reviews [11, 13] finding no effect. A recent meta-analysis [14] reported less risk of childhood asthma associated with cats, but increased risk with dogs. 

These disparate findings may be partly explained by inclusion of articles with different study designs. To date, there are no randomized controlled trials (RCTs) on the effect of pet exposure on allergic disease outcomes. In the absence of RCTs, the most valuable evidence is provided by longitudinal studies with a wealth of baseline data and frequent followups which enable assessment of pet exposure prior to the outcome of allergic disease. Despite this, all the current reviews have included at least two study designs (case control and cohort) [14] with the remainder also including cross-sectional studies [10–12]. 

Differences in the timing of exposures between studies may also provide a reason for varied results. It has been proposed that there are important windows of immune development [15] in which environmental exposures can either increase or decrease the risk of subsequent allergic disease development [16]. The perinatal period encompassing 20 weeks prior to birth until 4 weeks after is a critical time in developmental maturation of the immune system [17]. There is good evidence that the developing immune system in the fetus is susceptible to environmental influences and that immune development *in utero* is epigenetically regulated [17] with maternal exposures influencing the child's propensity for allergic disease [18, 19]. To date no reviews have limited assessment of pet keeping exposure at the critical perinatal period which may have a differential effect on risk than pet exposure at other periods of life. Lastly, another source of difference between studies is the varied study settings especially urban versus rural environment. A key relevant difference is the way in which pets are kept and the interaction between pets and other animals in rural settings. Hence, the clearest way to tease out the effects of cat and dog exposure on asthma and allergy in children would be to study this in an urban environment. None of the reviews have taken this into account.

 Therefore, we have conducted a systematic review of longitudinal studies in urban environments to explore the relationships between cat and dog exposure in the perinatal period and subsequent asthma or allergy.

## 2. Methods

### 2.1. Inclusion Criteria

- Human.
- Full-term infants.
- Population based or allergy enriched sample.
- Exposure to cat and/or dog presence or allergen levels measured and reported from 20 weeks prior to birth until 4 weeks after birth.
- Urban households only.
- Outcome assessed and reported—any allergic disease (asthma/wheeze/eczema/allergic rhinitis/food allergy) or atopy/sensitization as measured by serum IgE (total or specific) or on Skin Prick Testing.
- Longitudinal (cohort) studies.

The comparison groups were the children not exposed to pets within each study.

### 2.2. Search Strategy

We searched Medline using the following strategy in PubMed. The last search date was 17 May 2011.

- One or more allergic disease outcome term: Allergy and Immunology “[Mesh] OR “Hypersensitivity” [Mesh]) OR “Asthma” [Mesh] OR “Respiratory Sounds” [Mesh] OR “Rhinitis” [Mesh] OR “Eczema” [Mesh] OR “Dermatitis, Atopic” [Mesh] OR “Immunoglobulin E” [Mesh]) OR “Bronchial Hyperreactivity” [Mesh]) OR “Food Hypersensitivity” [Mesh] OR “Allergens” [Majr]

AND

(ii) One or more pet exposure term: “Pets” [Mesh] OR “Animals, Domestic” [Mesh] OR “Cats” [Mesh], OR “Dogs” [Mesh]

AND

(iii) One or more age of exposure term: “Prenatal Exposure Delayed Effects” [Mesh] OR “Maternal exposure” [Mesh], OR “Fetus” [Mesh], OR “Infant, Newborn” [Mesh] OR “Birth”

AND

(iv) Study type-cohort, NOT review.

### 2.3. Process for Selecting Studies

**Figure 1:**
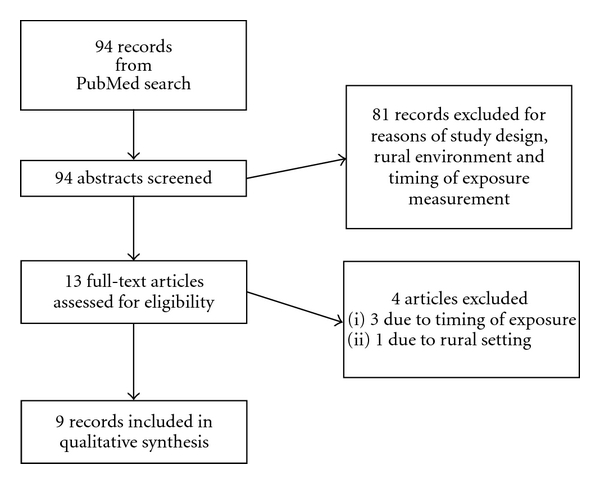
Flow chart of searching process.

A flow chart of the study selection process is shown in Figure 1. One author assessed all abstracts for eligibility. Full-text articles of eligible abstracts were then further assessed by the same author.

## 3. Results

Information concerning population, study type, exposure, outcome, and consideration of interaction by familial allergy for each of the nine studies is presented in Table 1.

The nine included articles represented 9 different studies. There were two articles included from one study, with outcomes presented at both 1 year [23] and 3 years [24] of age. Additionally one article reported on the findings of two studies [22]. Six studies were population based and three were on children at increased risk of allergic disease. The numbers analyzed ranged from 174 [26] to 2531 [27], while the total population included across all studies was 6,498. Only one article reported pet exposure exclusively as quartiles of allergen levels in vacuumed dust [21]; all other articles simply recorded the presence of cats and/or dogs in the home.

The allergic outcomes reported included: eczema in 4 articles; asthma/wheeze in 3; neonatal IgE in 3; sensitization in 2; allergic rhinitis in 1; food allergy in 1, and allergic symptoms (combination of eczema, asthma and hay fever) in one. The definitions and ages at which these outcomes were assessed varied between studies.

When assessing the quality of observational studies, particularly for possible sources of bias, the importance of addressing the following essential areas has been highlighted [29]: appropriate selection of participants, appropriate measurement of variables, and appropriate control of confounding. An assessment of the quality of included studies with reference to these areas is presented in Table 2.

Selection of participants and measurements of exposure and outcome variables were not thought to be sources of bias in any of the studies. None of the studies commented on recall bias although most of them recorded a parental history of allergy (a possible source of bias) retrospectively. Most of the studies used questionnaires for collecting data, so that interviewer bias was not applicable. Where interviews were performed however, whether or not the interviewer was blinded to the pet exposure status was not mentioned. There was possible bias due to loss to followup in four articles which did not explore whether those missing were different from those remaining in the study [22, 24, 26, 27]. Included confounders varied between studies introducing a possible source of bias.

The studies were divided into two groups based on when pet exposure was recorded. There were three studies which reported prenatal cat and dog exposure, and nine which reported exposure to cat and dog in the neonatal period. 

### 3.1. Exposure Recorded Prenatally [20, 21, 26] 

#### 3.1.1. Neonatal IgE

The main outcome from all three articles was the total level of neonatal IgE, measured from cord blood [20] or from the neonatal screening heel prick test at 3–5 days after birth [21, 26]. A lower level of IgE at birth was found in two articles [20, 26] if dogs [26], or cats or dogs [20] were kept during pregnancy. Both of these articles further restricted their analysis to a subgroup of low-allergy-risk infants and confirmed a lower level of neonatal IgE. The third article, which found no association [21] between neonatal IgE and pet exposure prenatally, was not strictly comparable having used levels of cat and dog allergens in dust as the measures of pet exposure.

#### 3.1.2. Sensitization

Additionally one of the articles [26], which was based on a selected allergy risk population, found a lower risk of IgE sensitization to cat at 12 months in those children whose mothers had been exposed to cats during pregnancy [26].

### 3.2. Exposure Recorded Postnatally [22–25, 27, 28]

There were six articles which recorded postnatal allergen exposure [22–25, 27, 28]. Although these articles recorded pet or pet allergen exposure in the first 4 weeks of life, almost all mothers would certainly have also had exposure during pregnancy. 

#### 3.2.1. Eczema

Four articles reported eczema as an outcome, variably defined as infantile eczema [27], atopic dermatitis [23, 24], or physician diagnosed eczema [22] and assessed at 6 months [27], 1 year [22, 23], or 3 years [24] of age. 

In three of these articles [23, 24, 27] (representing two studies), the risk of atopic dermatitis or infantile eczema was reduced for children exposed to dogs [23, 24] or pets [27] at birth. The Childhood Origins of Asthma Study (COAST) [23, 24] followed up a high-risk birth cohort and found a lower risk of atopic dermatitis by 1 year and at 3 years when exposed to dogs at birth. There was no association with cats. In an unselected population from Oslo [27], it was reported that pets at birth conferred less risk of infantile eczema by 6 months of age. When stratified by paternal atopy (asthma and hay fever) only the high-risk group still showed less atopic eczema risk.

In the other article [22] (representing two studies), the exposure groups were stratified by genetic/hereditary factors and the risk of eczema was increased only in the high-risk groups if exposed to a pet. Bisgaard et al. [22] identified children with either of 2 filaggrin- (FLG)-null mutations in both the Copenhagen Prospective Study on Asthma in childhood (COPSAC) and the Manchester Asthma and Allergy study (MAAS) birth cohorts. When exposed to cats at birth, children with a FLG null mutation had an increased risk of eczema in the first year: there was no convincing evidence for a similar relationship with dog exposure at birth. 

#### 3.2.2. Wheeze/Asthma

Three articles measured asthma or wheeze as an outcome [24, 27, 28]. The variables measured included current wheeze at age 3 years [24], bronchial obstruction or asthma at age 4 years [27], and frequent wheezing (>3 episodes per year) at ages 1–13 years [28]. All of these studies reported reduced odds or a reduced hazard ratio associated with a dog [24, 28] or with pets at birth [27]. No studies showed an association with cats alone.

Two of these three studies stratified by family history of allergy or parental asthma [27, 28], one [28] finding a risk reduction in the group without a parental history of asthma whilst the other found no change in risk [27].

#### 3.2.3. Other Outcomes (Sensitization, Rhinitis, Food Allergy, or Combined Variable)

There were three studies which measured sensitization as an outcome. Two of these showed no association [28, 30] with pet exposure at birth, whilst the third reported reduced allergen sensitization at 1 year in those children exposed to a dog at birth [23]. One article [27] examined allergic rhinitis at age 4 years and found a reduced risk in children exposed to pets at birth which persisted in both groups following stratification for parental atopy. 

Only one article reported on the outcome of food allergy. This study found no effect of cat or dog at birth on the risk of “confirmed food allergy” [23] (defined as specific IgE to egg milk or peanut of ≥0.35 kU/L and a convincing history). 

One article [25] reported “allergic symptoms” at 2 years of age (physician diagnosed eczema, chronic bronchitis or asthma, or hay fever) and found a reduction in symptoms among the children of families without a history of allergic disease if they had kept dogs at birth. However in dog-keeping families with a history of allergic disease, a modest increase was found.

#### 3.2.4. Summary of Results

Of the nine studies which were included, six [20, 23, 24, 26–28] found that the risk of allergic disease, or allergic sensitisation, was reduced in children who had been exposed to pets at or before birth. Dogs were associated with less risk of allergic outcomes in four [23, 24, 26, 28] of the six articles, while the remaining two found either pet to be associated with a reduced risk [20, 27]. Additionally one article reported a lower risk associated with cat exposure [26]. Only one small study (*n* = 174) failed to find an association [21]. 

Measures of familial allergy or pet avoidance were employed by four [20, 26–28] articles to account for the confounding aspects of pet-keeping choices. Limiting the analysis to the non-allergy-prone low-risk group did not change the associations in two articles [20, 26]. The remaining two articles [27, 28] stratified their analyses by familial high- and low-risk allergy groups. Subsequently, Nafstad et al. [27] found reduced odds of allergic disease in both high- and low-risk groups, whilst Remes et al. [28] found the reduced risk of allergic disease in pet-exposed children was limited to the low-risk familial allergy group.

This finding was further supported by the remaining two articles [22, 25] which only presented results stratified by allergic predisposition. Both found that high-risk children had an increased risk of allergic outcomes in the presence of dog or cat exposure at birth.

## 4. Discussion

Overall we found that for children without a family history of allergy, owning a dog was protective against the development of allergic disease. By contrast the findings with regard to those with a family history of allergy were more difficult to interpret. A major confounding factor may be that pet-keeping behaviour is likely to be strongly influenced by the allergic status of the parents and siblings, as is the child's risk of allergic disease. 

Further work assessing the impact of allergic status on owning a pet will be required to better understand whether this effect is due to a gene-environment interaction.

One of the problems in this field of research as outlined above is the inability to completely account for the confounding effects of pet-keeping choices made by allergic families. Families with allergic members are less likely to keep pets [31, 32], so it may appear that allergic disease is associated with not keeping pets. The lack of randomized controlled trials of pet-keeping, which would remove the confounding effect associated with pet-keeping choices in allergic families, makes the next best evidential study design a prospective birth cohort [33]. The ability of cohort investigators to manage the effects of confounding by familial allergy depends upon the validity and completeness of the information they have gathered concerning familial allergy and pet-keeping choices. 

In this systematic review, we found that the treatment of confounding by familial allergy status varied greatly between articles (Table 2). Some of the articles limited their analyses to low-risk groups, which gave only the less interesting half of the picture. There was no uniform measure of familial predisposition, with some articles using only parental asthma, some using all first degree familial allergic disease or atopy and one study combining this with pet avoidance behaviour. The varied nature of the measurement of familial allergic predisposition made it less likely that a clear picture of the relationship between pet-keeping and allergic disease would emerge. The articles also differed in which factors they included in their analytical models as potential confounders. 

Another problem with pooling studies for a systematic review is that they may measure outcomes at different ages. As part of a comprehensive systematic review, Chen et al. [11] grouped 21 birth cohort studies whose measured outcome of wheeze varied from 1 year [34] to 12 years [28]. The nature of wheeze at these two ages is very different. Over half of wheeze recorded in early childhood is transient [35–37]. Therefore, studies reporting wheeze/asthma outcomes in early childhood will identify many children who will not go on to have true asthma at school age and may be less specific in their findings than those which measure wheeze after six years of age. This was also an issue for our articles where three included articles measured wheeze at or before four years of age. 

Other issues related to the studies in this systematic review were the possibility of attrition bias due to loss of followup was not always explored, recall bias for survey questions related to parental allergies was not mentioned by any of the articles, and, reporting bias might also have influenced which articles were identified. Also, due to the perinatal exposure criterion, other birth cohort studies including the Multicentre Allergy Study [38], which measured pet exposure at 6 months, but not during the first month of life, were excluded.

Compared to previous reviews which have yielded inconsistent results, the strengths of this review are that it has measured pet exposure at one time period, the perinatal period which is arguably a critical exposure window for immune system maturation; it has included only one study design, the cohort study which is the design with the most evidential weight in this field; it has included only urban populations, thus avoiding other potentially confusing exposures present in rural communities; it has also identified the important role of familial allergy in interpretation of any results.

In the current systematic review, we were unable to perform a meta-analysis on the included articles due to heterogeneity in timing and assessment of exposures and outcomes. Despite this, the findings appear to have similarities across articles.

## 5. Conclusion

This paper of longitudinal studies of perinatal cat and/or dog exposure in urban populations suggests that dog exposure may have a protective effect on the risk of allergic disease in low-risk populations. Unfortunately in children at high-risk of allergic disease, there is still no clear answer. Further longitudinal studies or randomised controlled trials, in which the effect of familial allergy on pet-keeping choices is clearly explored, are needed.

## Figures and Tables

**Table 1:** 

Study group, authors' name and year of publication	Population and study type	Exposure variable(s) and prevalence of pet-keeping	Outcome variable(s)	Familial allergy stratification	Findings
Wayne County Health, Environment, Allergy and Asthma Longitudinal Study (WHEALS) Aichbhaumik, 2008 [20]	Unselected birth cohort in southeast Michigan Recruited women (21–49) in second trimester from Henry Ford Health System *N* = 1258 (1049 included in analysis)	*Pets *(*cats or dogs*) inside home at least 1 hr/day during pregnancy	*Cord blood IgE * *N* = 1049 (83%) Detection level ≥ 0.01 IU/mL	*Restricted * Excluded those families who selectively avoided pets because of allergy and still found reduced IgE in “low-risk” babies exposed to indoor pets	Infants in pet homes had decreased mean cord blood IgE 0.34 IU/L versus 0.24 IU/L *P* ≤ 0.001 (similar for cats and dogs separately) Cord IgE higher in atopic/allergic mothers Serum IgE already higher in mothers with no pets

Prevention of asthma in children (PREVASC) intervention trial, Schönberger, 2005 [21]	Control group only of interventional birth cohort *N* = 221 (174 included in analysis)	*Dust samples* collected in 4th to 6th months of pregnancy from maternal mattress and living room Fel d 1 and Can f 1 levels were divided into quartiles	Days 3–5 after birth—*heel prick blood IgE* (total) Dichotomized at 0.5 IU/mL	*No*	Proportion elevated IgE increased with increasing HDM but not cat or dog

Copenhagen Prospective Study on Asthma in Childhood (COPSAC) and Manchester Asthma and Allergy Study (MAAS) Bisgaard, 2008 [22]	COPSAC: neonates (1 month) of mothers with asthma Birth cohort *N* = 411 (353 included in analysis) MAAS: Unselected birth cohort *N* = 996 (412 included in the analysis)	*Pet at home * On interview: cat or dog living in house at birth	*Age of eczema * COPSAC: physician diagnosed based on Hanifin-Rajka criteria: 1, 6, and 12 months. MAAS: parental report, ISAAC questions at 1 yr	*Filaggrin mutations *Homozygosity or heterozygosity to 2 (R501X and 2282del4)	In both cohorts found: FLG genotype increased risk of eczema in first year HR 2.26 (95% CI 1.27–4.00) COPSAC in presence of cat this increased to HR 11.11 (3.79–32.6) In the filaggrin mutation group the HR for cat exposure was 7.49 (2.37–23.7) MAAS-3.82 (95% 1.35–10.8) (no increased risk in those without mutation) In the filaggrin group the HR for cat exposure was 2.47 (1.09–5.62) Dog exposure protective HR for COPSAC but increased risk for MAAS (not as marked as cat) In filaggrin loss of function variants there is no increase in eczema without the presence of a cat at birth

*Childhood Origins of Asthma Study (COAST) University of Wisconsin Gern 2004 [23]	Selected on basis of parental aeroallergen atopy or physician diagnosed asthma or both *N* = 312 families Birth cohort (285 included in analysis)	*Cat or Dog at home* at child's birth	*Atopic Dermatitis*: physician diagnosed, during first year or at 1 year *Sensitization * Allergen-specific IgE values ≥0.35 kU/L at 1 yr Allergens: egg, milk, peanut, Dust mite (*D*. *pteronyssinus* and *farina*e), and *Alternaria * *Food Allergy*: allergen-specific IgE + historical reports from parents or physician documentation	*No * Parental allergy and asthma considered as confounder, not formally modelled for interaction despite finding that fathers with cat allergy less likely to keep cats, and dog ownership more likely if mothers not cat allergic	Dog exposure at birth was associated with reduced allergen sensitization (19 versus 33%, *P* = 0.02) and atopic dermatitis at 1 year (30% versus 51%, *P* < 0.001) Cat exposure was not associated No association of cat or dog exposure on food allergy Postnatal exposure to dogs modified immune development by enhancing IL10 and IL13 responses. *P* values 0.12 and 0.08

*COAST Bufford 2008 [24]	Same cohort with followup at 3 years *N* = 312 Birth cohort (275 included in analysis)	*Cat or Dog at home* at child's birth	*Atopic dermatitis* or *wheezing* in the past year	*No*	Dog at birth associated with current AD OR 0.35 (0.15–0.83) at 3 years Current wheeze at 3 OR 0.49 (0.25–0.95) No association with cats

Mothers of German Nationality Pohlabeln 2008 [25]	Unselected mothers from 5 hospitals in 3 cities in northwest Germany Birth cohort *N* = 3132 (1881 included in analysis)	*Cats and dogs at home* at birth	ISAAC questions *Physician diagnosed eczema or itchy rash* for more than 6 months or cracked earlobes *Physician diagnosed asthma or chronic bronchitis * *Physician diagnosed hay fever * All these were considered as ever rather than current *Allergic symptoms:* any of eczema/asthma/hay fever defined above	*All Stratified * by first degree relatives with a history of allergic disease (reported history of asthma, eczema or hay fever in parents or siblings)	Newborns without a family history of allergic disease had a lower prevalence of asthma and eczema at age 2 when their families kept a dog. OR 0.52 (0.33–0.83) The risk was modestly elevated in allergic families who kept a dog in the allergic OR 1.43 (0.95–2.15) No associations found for cats or other pets

Prevention and Incidence of Asthma and Mite allergy (PIAMA) Kerkhof 2005 [26]	Selected: allergy high-risk birth cohort 1327 children of allergic mothers 2819 children of nonallergic mothers (1027 included in neonatal analysis, 492 included in 12 month, 682 included in 4 year)	*Pets at home in last trimester of pregnancy*	*Total IgE from heel prick* neonatal screening *Specific IgE at 12 months and at 4 years * ** (**HDM, cat, dog grass, milk, egg)	*Restricted * Stratified by “allergy status” of mother for reporting demographics but not analysed separately Also, excluded those families with pet avoidance or familial allergy—no change in associations (low-risk group only)	Dogs during pregnancy had less risk of high IgE at birth OR 0.5 (0.2–1.0) Cats during pregnancy and less cat sensitization at 12 months OR 0.6 (0.4–1.0)

Oslo birth cohort Nafstad 2001 [27]	Unselected 3754 children Birth cohort (2531 included in analysis)	*Pets in home at birth*	Survey assessed *bronchial obstruction, asthma, allergic rhinitis, and infantile eczema *	*Stratified *by parental atopy but found no change (parental atopy based on questionnaire concerning asthma and hay fever)	Less odds Asthma 4 yrs 0.7 (0.5–1.1) Allergic rhinitis 4 yrs 0.6 (0.4–1.0) Infantile eczema 6 months 0.7 (0.5–0.9)

Tucson Children's Respiratory Study (TCRS) Remes 2001 [28]	Unselected birth cohort—1246 healthy babies (1076 included in frequent wheeze analysis; SPT analysis included 737 (6 yrs), and 613 (11 yrs); IgE analysis included 829 (9 months), 534 (6 yrs), and 462 (11 yrs))	*Cat or dog at birth*	*Frequent wheezing*: >3 episodes of wheeze in the last year (from 1–13 years) *SPT at 6 and 11yrs * * Serum IgE at 9 months and 6 and 11* yrs (*alternaria*, HDM mix, Bermuda grass, careless weed, mesquite tree, mulberry tree, olive tree)	*Stratified * by parental history of asthma (asthma diagnosed in either parent)	Dogs at birth associated with less risk of developing frequent wheeze—seen only in children whose parents did not have asthma HR 0.47 (0.31–0.72) There was no increased risk in the parental asthma group No association with cats. Both nonatopic and atopic children with dogs at birth also had a reduced risk of wheeze. HR 0.47 (0.24–0.91) and HR 0.56 (0.32–0.98) Neither dog nor cat at birth associated with SPT positivity or IgE

^∗^These articles are both from the same study group.

Abbreviations: HDM: house dust mite; IgE: immunoglobulin E; SPT: skin prick Test.

**Table 2:** Evaluating the role of bias in the included studies.

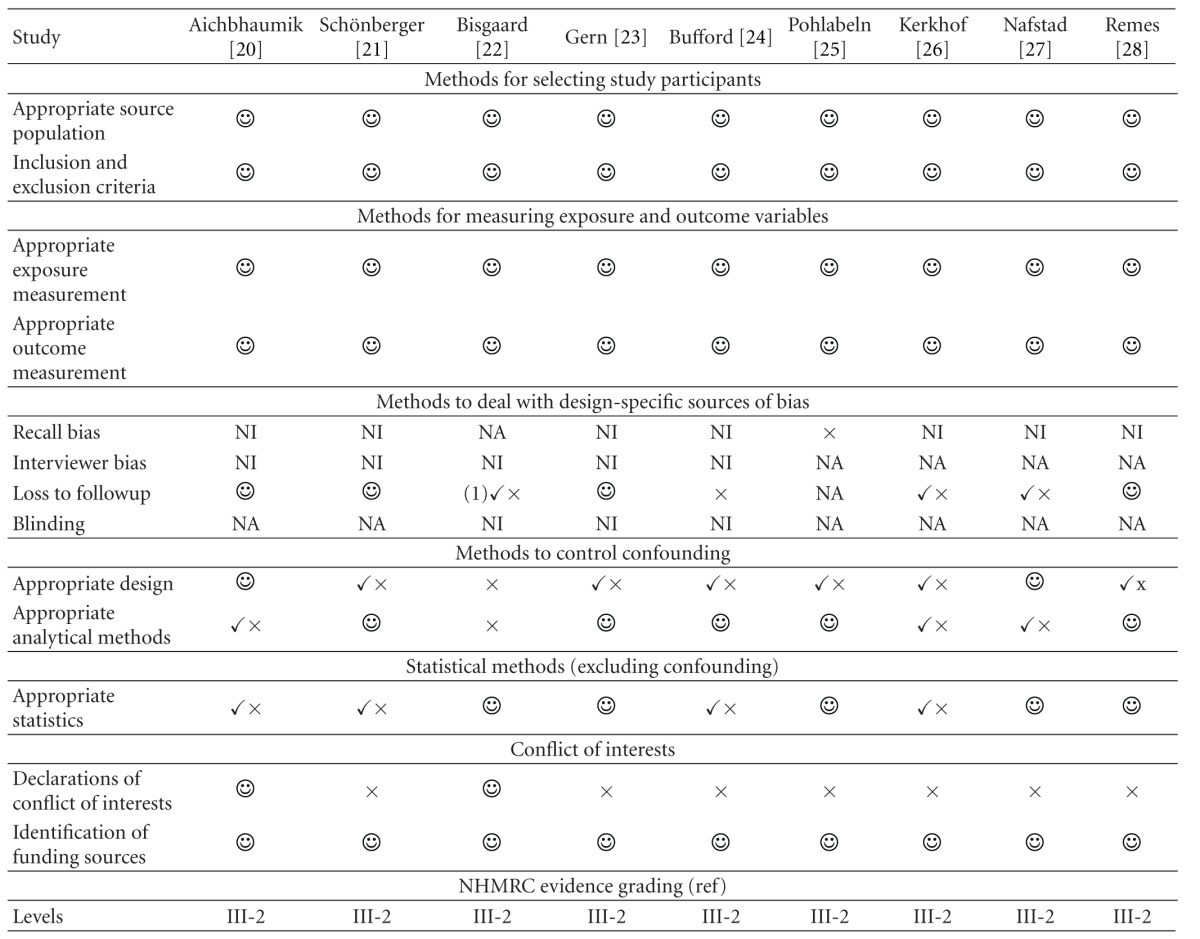

NA: not applicable; NI: not described in article.

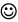
: adequate; *✓*×: poor; ×: not done.
